# Putting Chronic Disease on the Map: Building GIS Capacity in State and Local Health Departments

**DOI:** 10.5888/pcd10.120321

**Published:** 2013-06-20

**Authors:** Marie Lynn Miranda, Michele Casper, Joshua Tootoo, Linda Schieb

**Affiliations:** **Author Affiliations:** Michele Casper, Linda Schieb, Centers for Disease Control and Prevention, Atlanta, Georgia; Joshua Tootoo, School of Natural Resources and Environment, University of Michigan, Ann Arbor, Michigan.

## Abstract

Techniques based on geographic information systems (GIS) have been widely adopted and applied in the fields of infectious disease and environmental epidemiology; their use in chronic disease programs is relatively new. The Centers for Disease Control and Prevention’s Division for Heart Disease and Stroke Prevention is collaborating with the National Association of Chronic Disease Directors and the University of Michigan to provide health departments with capacity to integrate GIS into daily operations, which support priorities for surveillance and prevention of chronic diseases. So far, 19 state and 7 local health departments participated in this project. On the basis of these participants’ experiences, we describe our training strategy and identify high-impact GIS skills that can be mastered and applied over a short time in support of chronic disease surveillance. We also describe the web-based resources in the *Chronic Disease GIS Exchange* that were produced on the basis of this training and are available to anyone interested in GIS and chronic disease (www.cdc.gov/DHDSP/maps/GISX). GIS offers diverse sets of tools that promise increased productivity for chronic disease staff of state and local health departments.

## Introduction

Geographic information systems (GIS) are computer-based systems that allow users to capture, store, analyze, and display geographically referenced data. GIS-based techniques have been widely adopted and applied in the fields of infectious disease and environmental epidemiology; their use in chronic disease programs is relatively new (1,2). Geographic analysis enables users to explore and overlay data by location and to generate clear and accessible maps and data reports that can serve as powerful tools for project development, community outreach, and policy design (3,4). Public health organizations are beginning to recognize that the professionals they employ need to learn how to use the tools offered by GIS (5,6) to address and manage priorities (7–9).

The Division for Heart Disease and Stroke Prevention (DHDSP) at the Centers for Disease Control and Prevention (CDC) is a leader in the use of GIS, making US county-level morbidity and mortality data for heart disease and stroke widely available via the *Interactive Atlas of Heart Disease and Stroke* (http://apps.nccd.cdc.gov/DHDSPAtlas/) as well as other atlases and resources (www.cdc.gov/dhdsp/maps).

DHDSP is collaborating with the National Association of Chronic Disease Directors (NACDD) and the University of Michigan (UM) to enhance the capacity of state and local health departments to use GIS for surveillance and program development. These organizations designed and are implementing a training program tailored to the needs of chronic disease programs in state and local health departments.

This article describes key components of this GIS capacity-building project, key GIS skills for chronic disease surveillance programs, and ways state and local health departments applied their acquired GIS skills. Many of the training materials described in this paper, along with a collection of GIS resources, are publicly available on the *Chronic Disease GIS Exchange* (www.cdc.gov/dhsp/maps/gisx/).

## Structure of the GIS Training Program

A request for applications for the GIS training program is widely disseminated to state and local health departments. For the initial round of trainings, which took place in 2006, two health departments were selected to participate, with 5 or 6 selected in more recent rounds (a total of 19 state and 7 local health departments had participated as of the preparation of this report) (Box). Participating health departments receive GIS resources and training over 6 months. Training is available in 2 ways. The first consists of three 2.5-day, in-person training sessions at UM, where the learning environment creates opportunities for interaction across health departments as well as between trainees and instructors. The second way consists of three 2.5-day on-site, in-person training sessions hosted by the health department that was selected for the training. With both training approaches, support is provided during the intervals between training sessions.

**Box. State Health Departments That Participated in GIS Training**

**Table:** 

Arkansas Department of Health
Colorado Department of Public Health and Environment
Idaho Department of Health and Welfare
Iowa Department of Public Health
Indiana State Department of Health
Louisiana Department of Health and Hospitals
Maine Center for Disease Control and Prevention
Massachusetts Department of Public Health
Michigan Department of Community Health
Minnesota Department of Health
Mississippi State Department of Health
Montana Department of Public Health and Human Services
Nebraska Public Health
New Hampshire Division of Public Health Services
New York State Department of Health
North Carolina Division of Public Health
Utah Department of Health
Texas Department of State Health Services
Wisconsin Division of Public Health

## Key Components of Training Approach

This training program addresses the chronic disease priorities within each health department and seeks to enhance collaboration among chronic disease units. In their applications for training, health departments are required to 1) state their priorities, 2) demonstrate how GIS would support actions to address those priorities, and 3) demonstrate previous collaborations among chronic disease units. Each health department must also select a 4-member team from diverse chronic disease units to participate in the training and provide letters describing how units will work together to implement a GIS workflow.

In addition, each participating health department must select an expanded team. The core team members attend all training events and share responsibilities for developing GIS applications. The expanded team varies in size by health department and serves to connect the core training team to resources and management from existing local GIS expertise, information technology infrastructure, and individual chronic disease programs. Health department teams schedule regular joint meetings of their core and expanded teams.

GIS approaches require careful attention to systems (hardware and software) and resources (people, data, and methods). To ensure that health departments have the appropriate system support, a needs assessment is conducted for each of the selected health departments. The needs assessment within each health department includes evaluation of available equipment, network and information technology (IT) set-up, compatibility of equipment with project software requirements, and data availability. The assessment of resources covers existing GIS expertise in other health department units, potential challenges to the core team’s participation in trainings, and trainees’ expectations and familiarity with GIS. The needs assessment is based on data provided in the application for training, on the core and expanded teams’ questionnaires, and on the IT questionnaire. The needs assessment 1) directs the provision and support of software, 2) advances project development, and 3) catalyzes development of customized training modules. Existing infrastructure for GIS in health departments that have already received the training varied from advanced to minimal. Most health departments had sufficient hardware and software systems to run core GIS functions and support databases, graphics, and statistical software packages. The virtual workspace proved more challenging, revealing barriers to accessing common data in a shared server environment across multiple users. The degree to which individual users could perform GIS computing tasks and integrate data across multiple platforms and users varied widely.

Esri (Redlands, California, formerly Environmental Systems Research Institute) enjoys a broad base among established GIS users at the local and state levels of government. Training materials are developed to be compatible with the Esri ArcGIS Desktop v 10x. Although the acceptance and widespread use of this software make it a compelling choice for participants, there are several obstacles to providing access to this software for health department teams. First, Esri software licenses and support are expensive. Second, navigating past, existing, and planned software licensing agreements and arrangements can prove challenging. To address these issues, we negotiated with Esri to provide health departments with software grants for 1-year complimentary licenses for ArcGIS Desktop, ArcView level functionality, the Spatial Analyst extension, and the Network Analyst extension. The needs assessment determines the number and type of licenses provided to each health department.

During the training, we offer the following types of comprehensive support to help participants develop their GIS capacity:

- Self-guided exercises that are posted to a research portal monitored by GIS analysts from UM and CDC. These exercises reinforce live training content and allow participants to practice skills and techniques at their own pace.
- Monthly conference calls that create opportunities for health department teams to interact with each other and with training team staff on topics ranging from administrative to technical.
- Office hours (via the Internet) to encourage conversations between health department teams and GIS analysts with a minimum of 3 years of GIS experience. Topics covered during office hours center on common issues and questions arising from GIS projects. A weekly 1-hour virtual meeting allows participants to interactively address issues, exchange ideas, and solve problems with their specific projects.
- On-demand support, which includes telephone and screen-sharing sessions. Health department trainees can schedule a help appointment with training staff and CDC GIS analysts. These one-on-one interactions permit focused troubleshooting and individualized feedback.
- Web-based research portal (www.cdc.gov/DHDSP/maps/GISX/training/index.html) on which all training-related content is posted. The site supports topical discussions, as well as data- and file-sharing.

Hands-on activities associated with both live and web-based training content incorporate data relevant to chronic disease prevention at the state or local level. In addition to state and local data, 3 national data sets are used to familiarize trainees with GIS techniques and methods. These data sets are InfoUSA (Omaha, Nebraska), a commercial database for select retail business types; CDC Wide-ranging Online Data for Epidemiologic Research (WONDER) for mortality data; and US Census 2010/American Community Survey 2006–2010 for population and socioeconomic data. Using these data helps develop GIS skills while health department-specific data are acquired. Once teams develop key skills, they are equipped to process, georeference, display, and summarize their own state and local data sets with the support of training staff.

Training modules are informed by the needs assessment and tailored to respond to defined priorities of participating health departments. We employ short lectures on conceptual content, followed by much longer hands-on laboratory classes, followed by group discussion about what was learned during the laboratory classes. These sessions are reinforced with web-based content and parallel self-guided exercises. To maximize the impact of the training program, we are designing training module versions that can be offered entirely on the Internet.

Initially, participants are given map projects to complete between training sessions. These projects are designed to develop and maintain participants’ introductory GIS skills and teams. Subsequently, participants design their own projects. These projects are unique to the chronic disease priorities of health departments and require intermediate and advanced GIS skills. In developing these projects, the training staff takes on the role of consultants working in partnership with the health departments.

## Key GIS Skills and Capacities

We tracked the GIS capacities identified as being most relevant by the participants trained to date. These capacities were grouped into 3 GIS skill domains and provide the structure and focus of the 3 training modules; they are posted online (www.cdc.gov/DHDSP/maps/GISX/training/index.html):

### GIS I: Organizing principles and data display

The skills and key concepts included in this domain are basic software skills such as navigating the ArcGIS 10x user interface, opening and saving a project, connecting to folders, adding data, viewing metadata, and exporting output. This domain also includes an awareness of geographic data, organization strategies, logic for locational data, recognition of common formats and structures, basic processing (eg, table joins and projections), and creating map layouts.

### GIS II: Data management

The key skills included in this domain relate to data management and organization. These skills support fundamental GIS concepts that permit novice users to evaluate and organize spatial data. With the knowledge developed during the GIS II module, users can perform basic operations on both tabular and spatial data sets. This domain also emphasizes the special privacy and confidentiality concerns created by working with spatial data and offers strategies for compliance.

### GIS III: Data analysis

Key concepts and skills included in this domain relate to the exploratory analysis of spatial data. Trainees are taught to perform proximity-based analyses (eg, calculating service areas) and to overlay analyses that use spatial joins. Health department teams then apply these skills in spatial analyses driven by programmatic priorities, available data, and staff capacities. More advanced data processing concepts related to masking techniques for point data are also covered.

## Sample GIS Applications From Training Teams

Participants attain a high level of skill and perform sophisticated types of analyses (Figure). The map in Figure A was published in a 2008 report (10). This report is a result of collaboration between the Michigan Cardiovascular Alliance, Chronic Disease Epidemiology Section, and the Heart Disease and Stroke Prevention Unit at the Michigan Department of Community Health. The maps in that report depict geographic disparities in cardiovascular disease, a range of heart disease and stroke death rates, hospitalization rates, hospital locations, emergency medical system structure, health care resources, and other relevant programs within the state.

**Figure:**
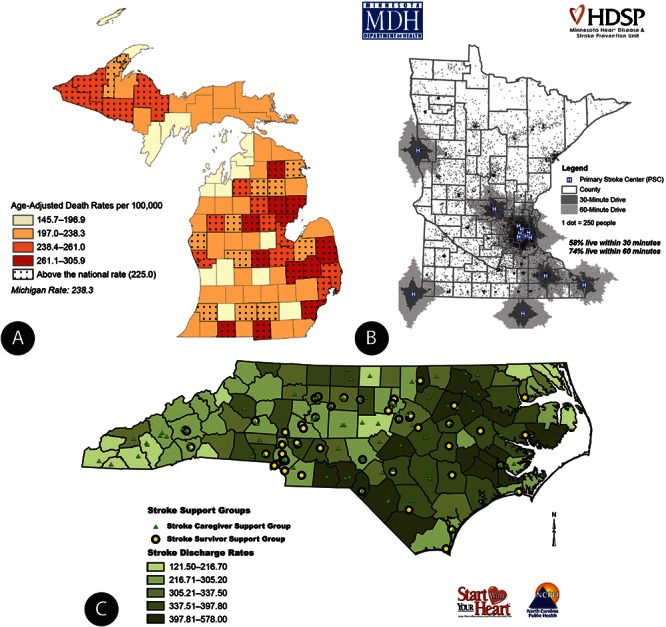
Examples of how state health departments applied their new GIS capacity to priority issues.

Michigan’s 2008 report on surveillance (10) was printed in color for wide distribution across the state, and both the report and individual maps may be downloaded from www.michigan.gov/cvh. The map in Figure A presents the age-adjusted heart disease death rate in Michigan by county. Counties that are speckled have an adjusted death rate above the national average of 225.0 per 100,000 people.

Figure B was developed by training participants from Minnesota. As a result of seeing this map, the Heart Disease and Stroke Prevention Unit at the Minnesota Department of Health started to assess the medical care infrastructure for the treatment of acute stroke emergencies. Certified primary stroke centers are committed to providing the highest level of stroke care, including the administration of tissue plasminogen activator (tPA), in-house neurology consultations, stroke care units, and inpatient and outpatient rehabilitation services. This map (available online at www.health.state.mn.us/divs/orhpc/rhac/presentations/033012.pdf) shows geographic areas within 30-minute and 60-minute drive times to 14 primary stroke centers in Minnesota and 6 in neighboring states. According to data from the 2010 Census, 70% of Minnesota’s population and 16% of Minnesota’s land area is within 60 minutes of one of these centers, but only 58% of the population is within 30 minutes. This map supported the work of a statewide stakeholder group called the Minnesota Acute Stroke System Council, which has approximately 100 members from more than 50 hospitals and organizations. The map has been incorporated into more than 20 presentations describing the need for a coordinated statewide acute stroke system (11). These presentations targeted a wide variety of external audiences, including practitioners at the annual Minnesota Stroke Conference, the Minnesota Rural Health Conference, stakeholders at the Minnesota Hospital Association, and Minnesota Emergency Medical Services Medical Directors. The illustration of service gaps and population coverage of existing certified centers created a demand for increasing the acute stroke care capabilities at all Minnesota hospitals, no matter their geographic location. The map is updated each time a new primary stroke center is designated and is included for the public on the Minnesota Stroke Partnership’s Data web page (http://www.mnstrokepartnership.org/mnstrokedata.html).

Figure C was developed by participants from North Carolina. In 2009, the Heart Disease and Stroke Prevention Branch at the North Carolina Division of Public Health identified various rehabilitation resources for stroke patients across the state. That information is included in *North Carolina Stroke Rehabilitation Programs and Services* (12). This map shows the locations of stroke support groups layered over the 2003–2007 stroke hospital discharge rates by county. The map is used by health departments to identify communities with the highest stroke rates and fewest resources, highlighting locations where partnerships may be formed to eliminate gaps in rehabilitation services for stroke patients.

## Discussion

This collaborative project, designed to address the gap in the use of GIS for chronic disease surveillance and program planning, demonstrates the feasibility and advantage of building GIS skills among public health professionals in state and local health departments. The key components contributing to the success of this project include 1) an emphasis on using GIS to address chronic disease priorities already established in each health department, 2) the collaboration of staff from multiple chronic disease units within each health department, 3) tailored exercises using state-specific chronic disease data for each health department, 4) multiple forms of readily available technical support, and 5) the selection of high-impact GIS skills to share with the participants. Together, these features enable trainees to integrate the use of GIS into decision-making processes within state and local health departments. Trainees were able to develop GIS skills quickly during the 4-month program and incorporate them into organizational operations and priority-setting.

The ability to design and produce their own chronic disease-related maps is especially important for state and local health department staff, given the current emphasis within CDC and state health departments on implementing a coordinated approach to chronic disease prevention and control (13). Maps can be used to address the 4 key actions outlined for this coordinated approach: 1) document communities with consistently heavy burdens of multiple chronic diseases, 2) examine environmental characteristics of those communities (eg, recreational and retail food environments, poverty), 3) study the distributions of existing prevention and treatment resources for chronic disease and issues regarding access to care, and 4) support improved community-clinical linkages (13).

Using GIS for chronic disease surveillance and program development supports an evidence-based approach to public health that maximizes population health (14). Outcomes from community assessments, a critical first step in an evidence-based approach, can be more effectively communicated to planners, partners, decision makers, and other stakeholders by using maps rather than graphs and tables. State and local health departments have shared their chronic disease maps with partners to identify common goals and strategies; they designed those maps to 1) serve as a basis for decision making by senior management within health agencies and legislative bodies and 2) facilitate collaboration among chronic disease units within agencies.

To maximize the opportunities for health department staff to learn how to use GIS, tutorials developed through this collaborative project are available online at the Chronic Disease GIS Exchange hosted by the Division for Heart Disease and Stroke Prevention at CDC (www.cdc.gov/dhdsp/maps/gisx). These tutorials include presentation slides used during trainings, hands-on exercises, and chronic disease data sets. The website also includes a gallery of chronic disease maps submitted by agencies from across the country and a selection of GIS references and resources.

In summary, the collaboration among CDC, NACDD, and UM has developed an engaging and effective approach for enhancing the use of GIS for chronic disease surveillance and program development within state and local health departments. This enhanced capacity provides staff in the participating state and local health departments with a powerful set of tools to efficiently and innovatively improve the health of populations in their jurisdictions.
